# Supplementary material for: Ophthalmic and Genetic Features of Bardet Biedl Syndrome in a German Cohort
Source: Genes (Basel). 2022 Jul 8;13(7):1218. doi: 10.3390/genes13071218 (PMC9322102; doi:10.3390/genes13071218)
Supplement: Supplementary file 1 [file genes-13-01218-s001.zip › Supplementary Table S4_Ophthalmology_Gene.pdf]

Patient ID	Age at Diagnositic Exam (yr)	Visual field (III4e)	Visual acuity OD	Visual acuity OS	Spherical Equivalent OD	Spherical Equivalent OS	ERG	flash VEP	AO performed	OCTR/L	AF (perimacular hyperfluorescent ring)R/L	Fundus R/L	First symptoms	Age of onset	Nystagmus	Strabismus	P15	Cataract
**BBS1: 15 patients**																		
BBS79	39	not recordable	0.02	0.04	-1.5	-1.75	extinguished	-		loss of photoreceptor layer, ILM wrinkling	no	RP	night blindness	childhood	no	no	total color discrimination impairment	RE/LE: IOL at age of 31
BBS49	16	RE/LE: concentric constriction 8-10°	0.02	0.02	-	-	extinguished	-		loss of photoreceptor layer, deposits adjacent to Bruch's membrane, foveal atrophy	yes	RP	night blindness, light photophobia	childhood	no	no	total color discrimination impairment	at age of 15 (sucapsular)
BBS30	34	RE/LE: 3-4° at age of 34	0.1	0.08	-	-	extinguished	good amplitude		loss of photoreceptor layer, foveal atrophy, ONL thinning	yes	RP	night blindness	19 yr	no	no	total color discrimination impairment	no
SRP17	46	not recordable	HM	HM	-	-	extinguished	severe reduction in amplitude		loss of photoreceptor layer, deposits adjacent to Bruch's membrane, ILM wrinkling	yes	RP	night blindness	childhood	no	yes, LE OP	total color discrimination impairment	RE/LE: at age of 39
BBS10-I	32	RE/LE: 2°	0.2	0.25	-1.75	-2	extinguished	good amplitude	yes	loss of photoreceptor layer, foveal atrophy	yes	RP	night blindness	childhood	no	no	total color discrimination impairment	IOL at age of 26
BBS10-II	26	RE/LE: 5°	0.25	0.05	-0.25	-0.5	extinguished	pattern VEP: severe reduction in amplitude	yes	loss of photoreceptor layer, deposits adjacent to Bruch's membrane, ILM wrinkling	yes	RP	night blindness	childhood	no	yes, LE OP	total color discrimination impairment	RE/LE: at age 19 (sucapsular)
BBS66	34	not recordable	0.03	0.03	2	1.5	extinguished	-		loss of photoreceptor layer, deposits adjacent to Bruch's membrane, ILM wrinkling	yes	cone-rod dystrophy	light photophobia, night blindness	6 yr	no	no	total color discrimination impairment	no
BBS18	43	not recordable	HM	HM	-	-	extinguished	severe reduction in amplitude		loss of photoreceptor layer, foveal atrophy, ILM wrinkling	yes	RP	night blindness	10 yr	yes	no	total color discrimination impairment	at age of 34 (sucapsular), IOL
BBS80	14	RE/LE: 5-7 °	0.05	0.05	0.5	0.5	extinguished	good amplitude		loss of photoreceptor layer, deposits adjacent to Bruch's membrane, internal limiting membrane wrinkling	yes	RP	night blindness	childhood	no	no	total color discrimination impairment	no
ARRP328	56	not recordable	HM	HM	-	-	extinguished	-		loss of photoreceptor layer, severe macular atrophy, ILM wrinkling (epiretinal membrane)	yes	RP	light photophobia	14 yr	yes	yes	total color discrimination impairment	RE/LE: IOL at age of 40
BBS87	15	RE:5-7°, LE:6- 7°	0.2	0.16	-7	-8.75	extinguished	good amplitude		loss of photoreceptor layer, foveal atrophy, ILM wrinkling	no	RP	night blindness	4 yr	no	no	Normal	no
BBS64	18	Perimetrie 90°: 50° nasal, superior, 70° temporal und 60° inferior(good function in periphery)	0.01	0.05	-4.75	-3.5	extinguished	good amplitude		persistence of the photoreceptor layer cells outside the fovea	yes	cone dystrophy (age 18)	light photophobia	7 yr	no	no	Normal	no
BBS78	30	not recordable	0.02	0.025	4.5	3.5	extinguished	-		loss of photoreceptor layer, ILM wrinkling	yes	RP	night blindness	childhood	yes	no	not possible	at age of 30
BBS54	19	RE/LE: 6- 8°	0.05	0.05	1	1	extinguished	reduction in amplitude		loss of photoreceptor layer, foveal atrophy, ILM wrinkling	yes	rod-cone dystrophy	night blindness, nystagmus	1 yr	yes	no	total color discrimination impairment	no
BBS77	11	not recordable	0.25	0.25	4	3.5	extinguished	-		loss of layer of photoreceptor cells outside the fovea, ONL thinning	no	cone-rod dystrophy	light photophobia, night blindness	childhood	no	no	not possible	no
**BBS2: 3 patients**																		
BBS53	21	not recordable	0.125	0.1	-3	-3	extinguished	-		loss of photoreceptor layer, foveal atrophy, internal limiting membrane wrinkling	no	RP	night blindness, light photophobia	childhood		childhood	total color discrimination impairment	R/L: IOL at 20 age
BBS35	25	RE/LE: 5-7°	0.1	0.2	-3.25	-3.5	extinguished	-		loss of photoreceptor layer outside the macula, internal limiting membrane wrinkling	yes, bull`s eye	cone-rod dystrophy	light photophobia, night blindness	6 yr	no	yes	total color discrimination impairment	R/L: at age of 25
ARRP379	8	not recordable	0.4	0.5	0.4	0.5	extinguished	-		loss of photoreceptor layer outside tha macula, internal limiting membrane wrinkling	no	cone-rod dystrophy	light photophobia	5 yr	no	no	not done	no
**BBS3: 3 patients**																		
BBS44-I	23	RE/LE: 30°	0.125	0.125	-4.5	-4	remaining scotopic response	-		loss of photoreceptors in fovea with good layer of photoreceptors towards periphery	yes	cone dystrophy	light photophobia	childhood	no	no	total color discrimination impairment	no
BBS44-II	13	central scotome ou	0.25	0.25	-6.75	-7.25	remaining scotopic response	-		loss of photoreceptor layer towards periphery	yes	cone-rod dystrophy	visual acuity imparment, light photophobia	childhood	no	no	total color discrimination impairment	no
BBS61	17	not recordable	0.01	0.05	-12.5	-10.5	extinguished	good amplitude		LE: loss of photoreceptor layer outside the fovea, internal limiting membrane wrinkling	no	cone-rod dystrophy	night blindness, nystagmus	1 yr	yes	yes, RE	total color discrimination impairment	at age of 17 yrs (polar)
**BBS4: 3 patients**																		
BBS37	23	not recordable	HM	HM	-3	-3.25	extinguished	-		loss of photoreceptor layer, foveal atrophy, internal limiting membrane wrinkling	yes	RP	night blindness	childhood	yes	yes	total color discrimination impairment	no
BBS45	30	not recordable	0.02	0.05	-8.5	-9	extinguished	reduction in amplitude		loss of photoreceptor layer, foveal atrophy, internal limiting membrane wrinkling	yes, bull`s eye	RP	night blindness	childhood	yes	no	total color discrimination impairment	R/L: at age of 27 (polar)
BBS55	22	not recordable	0.006	0.06	-10	-10	extinguished			LE: loss of photoreceptor layer, foveal atrophy	yes	RP	night blindness, nystagmus	1 yr	yes	childhood	total color discrimination impairment	at age of 18
**BBS5: 3 patients**																		
BBS56-I	20	RA about 4°, LA about 3°, peripheral temporal remning islands at age of 20	0.16	0.16	-0.75	-0.75	extinguished	Pattern VEP: severe reduction in amplitude	yes	loss of photoreceptor cell layer outside the fovea, internal limiting membrane wrinkling	yes	rod-cone dystrophy	night blindness	childhood	yes	yes	total color discrimination impairment	at age of 23 (subcapsular)
BBS56-II	7	not recordable	0.5	0.1	-3.5	-1	NP	-		loss of photoreceptor cell layer outside the fovea, internal limiting membrane wrinkling	no	rod-cone dystrophy	visual acuity impairment at age 3 yr		no	no	not possible	no
BBS43	37	not recordable	NLP	NLP	-	-	extinguished	-		RE: severe loss of photoreceptor layer	yes at age 37 yr	RP	night blindness	10 yr	yes	no	not possible	no
**BBS6: 1 patient**																		
BBS33	48	not recordable	0.05	NLP	-8.5	(prothesis)	extinguished	-		severe loss of photoreceptor layer in RE	no	RP	light photophobia, night blindness	childhood	yes	LE: enuculation	not possible	R/L: IOL at age of 26
**BBS7: 3 patients**																		
BBS70	20	RE/LE: 10°	0.07	0.05	1.5	1	extinguished	good amplitude		loss of photoreceptor layer outside the fovea	yes	RP	night blindness, light photophobia	childhood	no	no	total color discrimination impairment	at age of 20 (polar)
BBS62	17	not recordable	0.328	0.32	-4.25	-5	extinguished	Pattern VEP: good amplitude		loss of photoreceptor layer, foveal atrophy, internal limiting membrane wrinkling	yes, bull`s eye	RP	night blindness, light photophobia	childhood	no	no	normal	no
BBS60	17	not recordable	0.04	0.06	3	2.5	extinguished	good amplitude		loss of photoreceptor layer, foveal atrophy, internal limiting membrane wrinkling	yes, bull`s eye	RP	light photophobia	12 yr	yes	yes	total color discrimination impairment	at age of 17 (polar)
**BBS8: 1 patient**																		
BBS67	17	RE/LE: 20-25°	0.125	0.05	3	3	extinguished	-	yes	loss of photoreceptor layer, foveal atrophy, internal limiting membrane wrinkling	yes	RP		6 yr	yes	yes, LE	not possible	no
**BBS9: 5 patients**																		
BBS40-I	43	not recordable	LS	LS	-5.25	-5.5	extinguished	severe reduction in amplitude		LE: loss of photoreceptor layer, foveal atrophy, internal limiting membrane wrinkling	no	rp	night blindness	childhood	yes	yes, RA	total color discrimination impairment	R/L: Keratoconus in RE
BBS42-I	30	not recordable	HM	CF	1.25	2	extinguished	-		loss of photoreceptor layer, foveal atrophy	yes, bull`s eye	RP	night blindness, light photophobia		yes	yes	total color discrimination impairment	at age of 22
BBS42-II	20	RE/LE: peripheral remaining islandsl	0.25	0.25	1	1.25	extinguished	-		loss of photoreceptor layer, foveal atrophy	yes, bull`s eye	rod-cone dystrophy	night blindness, light photophobia	childhood	yes	yes	total color discrimination impairment	R/L: at age of 15
LCA70	39	not recordable	LS	LS	-1.5	-1	NP	-		loss of photoreceptor layer, foveal atrophy	yes, bull`s eye	RP	night blindness, visual acuity impairment at age 10 yr	childhood	yes	yes	not possible	no
BBS74	6	not recordable	0.63	-0.3	-3.25	-3.25	extinguished	-		NP	no	cone-rod dystrophy	visual acuity impairment with amblyopia since first year, night blindness at age 3 yr	childhood	no	no	total color discrimination impairment	no
**BBS10: 20 patients**																		
BBS81	11	RE:3-5°.LE:7-10°	0.25	0.2	-4	-4	extinguished	pattern VEP: severe reduction in amplitude		loss of photoreceptor layer, fovea atrophy, ILM wrinkling	yes	RP	night blindness	2 yr	until age of 5	yes	total color discrimination impairment	no
BBS48	38	not recordable	LP	LP	-	-	extinguished	-		loss of photoreceptor layer, foveal atrophy, ILM wrinkling	yes	RP	night blindness, light photophobia	childhood	yes	yes	total color discrimination impairment	RE/LE: at age of 20
BBS46	34	poor fixation	0.06	0.06	-2	-2.25	extinguished	-		loss of photoreceptor layer, foveal atrophy, ILM wrinkling	yes	RP	night blindness	10 yr	yes	no	total color discrimination impairment	at age of 30 (polar)
BBS16	37	not recordable	0.02	0.02	-3	-1.25	extinguished	good amplitude		loss of photoreceptor layer, foveal atrophy	no	rod-cone dystrophy	night blindness	childhood	yes	no	total color discrimination impairment	at age of 29 yr (polar)
BBS83	23	not recordable	0.02	LS	-2.25	-2.5	extinguished	good amplitude		loss of photoreceptor layer, foveal atrophy, ILM wrinkling	yes	RP	night blindness, light photophobia	childhood	yes	yes	total color discrimination impairment	at age of 21
BBS84	5	not recordable	0.4	0.2	3.25	4.75	extinguished	-		loss of photoreceptor layer outside the fove	yes	cone-rod dystrophy	visual acuity impairment with amblyopia since first year then nightblindnes at age of	polydactyl at birth, at age 2 years clinical	no	yes	total color discrimination impairment	no
BBS13	28	no fixation, nystagmus, not recordable	HM	0.1	-5	-3.5	extinguished	good amplitude		loss of photoreceptor layer, fovea atrophy, ILM wrinkling	yes	RP	night blindness	childhood	yes	no	total color discrimination impairment	RE/LE: IOL at age of 26
BBS85	13	RE/LE: 12-15°	0.3	0.3	-2.5	-2.5	extinguished	-	yes	loss of photoreceptor layer outside the fovea, ILM wrinkling	NP	RP	strabismus at age 1 yr	childhood	no	yes, RE	not possible	no
BBS75	7	not recordable	0.25	0.25	-5.25	-4.75	extinguished	-	yes	loss of photoreceptor layer outside the fovea, ILM wrinkling	yes	cone-rod dystrophy	night blindness	childhood	no	no	total color discrimination impairment	no
BBS15	6	RE/LE: 1-2°	0.05	HM	-3.25	-3.25	extinguished	-		NP	yes, at age 25 yr	RP	night blindness	childhood	yes	no	total color discrimination impairment	no
BBS76	24	RE/LE: concentric constriction 10° Grad	0.125	0.125	-4.5	-5.5	extinguished	-		loss of photoreceptor layer, fovea atrophy, ILM wrinkling	yes	cone-rod dystrophy	night blindness	6 yr	no	no	total color discrimination impairment	at age of 24
BBS82	12	semiautomatisierte kinetische Perimetrie: R/L concentric constriction superior 10°, nasal 25°, inferior 45°,	0.2	0.03	1	0.5	extinguished	reduction in amplitude		loss of photoreceptor layer, fovea atrophy,ILM wrinkling	yes	rod-cone dystrophy	night blindness, light photophobia	3 yr	yes	no	total color discrimination impairment	no
BBS72	41	not recordable	amaurosis	amaurosis	-	-	extinguished	-		loss of photoreceptor layer, foveal atrophy,ILM wrinkling	no	RP	light photophobia, night blindness	childhood	no	no	not done	R/L: IOL at age of 38
SRP95	41	not recordable	LP	HM	-	-	extinguished	reduction in amplitude		severe atrophy of photoreceptor layer	NP	RP	night blindness	childhood	yes	yes	total color discrimination impairment	at age of 29 (polar)
BBS50	31	not recordable	HM	HM	1.5	2.25	extinguished	-		loss of photoreceptor layer, foveal atrophy, ILM wrinkling	yes	RP	night blindness, light photophobia	childhood	yes	no	protan-axis defect	at age of 31 (subcapsular)
BBS71	9	not recordable	0.02	0.1	-0.5	-0.5	extinguished	-		loss of photoreceptor layer, foveal atrophy, ILM wrinkling	no	RP	light photophobia, night blindness	2 yr	yes	no	not possible	no
BBS86	9	not recordable	0.125	0.1	2	2	extinguished	-		loss of photoreceptor layer, foveal atrophy, ILM wrinkling	NP	cone-rod dystrophy	night blindness, strabismus at age 2 yr	2 yr	yes	yes, LE	total color discrimination impairment	no
BBS47	36	not recordable	LS, LL	LS, LL	-5	-5.5	extinguished	-		loss of photoreceptor layer, foveal atrophy, ILM wrinkling	no	RP	night blindness	childhood	yes	no	total color discrimination impairment	R/L: at age of 31
BBS57	19	not recordable	0.02	0.06	-2.5	-2	extinguished	good amplitude		LE: loss of photoreceptor layer, foveal atrophy, ILM wrinkling	no	RP	night blindness	childhood	yes	no	total color discrimination impairment	no
RCD768	29	RE/LE: concentric constriction with beginnings of ring scotoma, central scotoma 4°	0.05	0.05	0.5	0.5	remaining scotopic response	-		foveal atrophy with persistence of the layer of photoreceptor cells outside the fovea	yes	cone-rod dystrophy	light photophobia	14 yr	no	no	total color discrimination impairment	no
**BBS12: 3 patients**																		
BBS59	21	relative central scotoma	0.8	0.63	0.8	0.63	scotopic response	-		An intact photoreceptors layer except for a small loss in fovea area (cone dystrophy).	yes	cone dystrophy	light photophobia,Night blindness	17 yr	no	no	normal	no
BBS58	16	Perimetry 90°: 50° nasal, superior, 70° temporal und 60° inferior (good function in periphery)	0.4	0.3	-5	-4	scotopic response	-		little loss of photoreceptors in fovea with good layer of photoreceptors outside towards periphery	yes	cone dystrophy (aged 16), peripapillary myopic conus	visual acuity impairment	11 yr	no	no	normal	no
BBS41	26	not recordable	HM	LS	-3.5	-3.5	extinguished	-		severe loss of the layer of photoreceptors	yes	rod-cone dystrophy	night blindness	childhood	yes	yes	total color discrimination impairment	R/L: at age of 26
**BBS16: 1 patient**																		
SRP890	42	RE/LE: 2-3° with large remaining islands nasal, inferior	0.32	0.4	0.5	0.5	extinguished	-		loss of photoreceptor layer outside the fovea, ILM wrinkling	no	RP	night blindness, light photophobia	16 yr	no	no	total color discrimination impairment	R/L: IOL at age of 34
