# Supplementary material for: Ophthalmic and Genetic Features of Bardet Biedl Syndrome in a German Cohort
Source: Genes (Basel). 2022 Jul 8;13(7):1218. doi: 10.3390/genes13071218 (PMC9322102; doi:10.3390/genes13071218)
Supplement: Supplementary file 1 [file genes-13-01218-s001.zip › Supplementary Table S5_systemic findings-Gene.pdf]

Patient ID	Polydactyly	Brachydactyly	Obesity	Hypogonadismus	Hirschspurg	Liver: malformations - fibrosis	Kidney dysfunction	Diabetes	Hypertention	Heart	Neurology/ Ataxia	Hearing disturbances	Speach difficulties	Anosomy	Development delay	Mental retardation	Behavioral problems	Asthma	Gout	Neurodermitis	Metformin 500 mg dayly	Vit D deficiency	Thyroid dysfunction
**BBS1: 15 patients**																							
BBS79	1 foot	no	no	unknown	no	no	yes	no	no	no	migraine	no	yes until age 6	no	yes	yes	no	no	no	no	no	no	no
BBS49	yes, 2 hands	yes	no	unknown	no	no	no	no	no	no	no	no	no	no	no	no	no	no	no	no	no	no	hypofunction
BBS30	2 hands and 2 feet	yes	yes	unknown	no	no	no	no	yes	no	no	no	no	no	no	no	no	yes	no	no	no	no	no
SRP17	no	syndactyly	yes	unknown	yes	no	no	no	yes	no	migraine	no	no	no	no	yes	no	no	no	no	no	no	no
BBS10-I	left hand and left foot	no	yes, until age 13	unknown	no	no	no	no	no	no	no	no	no	no	no	no	no	no	no	no	no	no	no
BBS10-II	2 hands	yes	yes	unknown	no	no	no	no	no	no	no	no	yes until age 5	no	yes	no	no	no	no	no	yes	no	hypofunction
BBS66	1 hand	yes	yes	no	yes	no	cystitis	yes at age 32, type 2	no	no	no	no	yes	no	no	yes	no	no	no	no	yes	no	no
BBS18	left hand and right foot	yes	yes	unknown	no	no	yes	no	yes	thrombosis in lung, once	no	no	yes	no	no	no	no	no	no	no	no	yes	no
BBS80	2 hands	yes	yes	unknown	no	no	no	no	no	no	no	no	no	no	no	no	no	no	no	no	no	no	no
ARRP328	no	no	yes	unknown	no	no	no	yes at age 36	yes	no	no	no	no	no	no	no	no	no	no	no	yes	no	hypofunction
BBS87	1 foot	no	yes	unknown	no	no	no	no	no	no	no	no	no	no	yes	no	no	no	no	no	yes	no	hypofunction
BBS64	no	yes	yes	unknown	no	fat	no	yes	no	no	no	no	no	no	no	no	no	no	no	no	yes	no	hypofunction
BBS78	2 hands and 2 feet	yes	yes	no	no	no	no	no	yes	no	no	no	no	no	no	no	no	no	no	no	no	no	no
BBS54	right foot	yes	yes	unknown	yes	yes	yes	no	yes	no	no	no	no	no	yes	no	no	no	no	no	yes	yes	no
BBS77	2 feet	yes	yes	no	no	no	no	no	no	no	no	no	no	no	yes	yes	no	no	no	no	no	yes	no
**BBS2: 3 patients**																							
BBS53	two feet	yes	yes	unknown	no	no	no	no	no	no	no	no	no	no	no	no	no	no	no	no	no	no	no
BBS35	yes, two feet	no	yes	no	no	no	small kidney	no	no	no	no	no	yes, until age 13	no	no	no	no	no	no	no	no	yes	no
ARRP379	no	no	yes	no	no	no	no	no	no	no	no	no	yes, until age 4	yes	no	no	no	no	no	yes	no	no	no
**BBS3: 3 patients**																							
BBS44-I	two feet	no	no	unknown	no	no	no	no	no	no	no	no	no	no	no	no	no	no	no	no	no	no	no
BBS44-II	two feet	no	no	unknown	no	no	no	no	no	no	no	no	no	no	no	no	no	no	no	no	no	no	no
BBS61	2 hands and 2 feet	yes	yes	yes	no	no	no	yes, insulin pump since age 10	no	no	no	no	no	no	yes	no	no	no	no	no	no	no	no
**BBS4: 3 patients**																							
BBS37	no	no	no	unknown	no	no	no	no	no	no	no	no	no	no	no	no	no	no	no	no	no	no	no
BBS45	2 hands and 2 feet	yes	yes	unknown	no	no	no	no	no	no	no	no	no	no	no	no	no	no	no	no	no	no	no
BBS55	2 feet and left hand	syndactyly	yes, since age 5	yes	no	no	no	no	no	no	no	no	yes, until age 5	no	yes	yes	no	yes	no	no	no	yes	hypofunction
**BBS5: 3 patients**																							
BBS56-I	right hand	no	yes	unknown	no	no	no	no	no	no	no	no	no	no	yes	yes	no	yes, until age 4	no	no	yes	yes	yes
BBS56-II	two hands	no	yes	unknown	no	no	no	no	no	no	no	no	no	no	no	no	no	no	no	no	no	no	no
BBS43	two feet	no	yes	no	no	no	no	no	no	no	no	no	no	no	yes	yes	no	no	no	no	no	no	no
**BBS6: 1 patient**																							
BBS33	2 hands and 2 feet	yes	no	yes	no	no	yes	no	yes	no	no	no	no	no	no	no	no	no	no	no	no	no	no
**BBS7: 3 patients**																							
BBS70	2 hands	no	yes since 2005	yes	no	no	no	no	no	no	no	no	no	no	no	no	no	no	no	no	no	no	no
BBS62	2 hands and 2 feet	yes	yes	unknown	no	no	no	no	no	no	no	no	no	no	yes	no	no	no	no	no	yes	yes	no
BBS60	2 hands and 2 feet	yes	yes	unknown	no	no	no	no	no	no	no	no	yes, until age 3	no	no	yes	yes	no	no	no	no	no	no
**BBS8: 1 patient**																							
BBS67	2 hands and 2 feet	yes	yes	no	no	no	no	no	no	no	no	no, tympanostomy tube	no	no	yes	yes	no	no	no	no	no	yes	no
**BBS9:5 patients**																							
BBS40-I	2 feet and left hand	keratoconus RA	yes	unknown	no	no	no	no	no	no	no	no	no	no	yes	yes	no	yes	no	no	no	yes	no
BBS42-I	right hand and left foot	no	yes	unknown	no	no	yes	no	yes	no	no	no	no	no	no	yes	no	yes, in childhood	no	no	yes	no	hypofunction
BBS42-II	one foot	no	yes	unknown	no	no	no	no	no	no	no	no	no	no	no	no	no	yes, in childhood	no	no	yes	no	hypofunction
LCA70	2 hands and 2 feet	yes	yes	no	no	no	yes	yes	yes	no	no	no	no	no	yes	yes	no	no	no	no	yes	?	no
BBS74	left foot	yes	yes	unknown	no	no	no	no	no	no	no	no	no	no	yes	yes	no	no	no	no	no	no	no
**BBS10: 20 patients**																							
BBS81	2 feet and left hand	yes	yes	unknown	no	no	yes	no	no	no	no	no	yes	no	yes	no	no	no	no	no	yes	no	no
BBS48	2 feet	no	yes	unknown	no	no	no	no	yes	no	no	no	no	no	no	no	no	no	no	no	no	yes	no
BBS46	right hand and right foot	no	no	yes	no	no	no	no	yes	no	no	no	no	no	no	no	no	yes	no	no	no	no	no
BBS16	2 hands and 2 feet	yes	yes	unknown	no	no	yes	no	no	no	no	no	no	no	yes	no	no	no	no	no	no	no	no
BBS83	2 hands and 2 feet	yes	yes	unknown	no	yes	no	no	no	no	no	no	no	no	no	no	no	no	no	no	yes	yes	hypofunction
BBS84	yes, 2 feet	yes	yes	unknown	no	hypercholesterolemia	small kidney	no	no	no	no	no	yes	no	no	no	yes	no	no	no	no	yes	no
BBS13	yes	no	yes	unknown	no	no	yes	no	no	no	no	yes until 7 years old	no	no	yes	no	yes	no	no	yes	no	no	no
BBS85	right foot	yes	yes	cyst in ovary	no	gall bladder removed	suspected cysts	no	no	no	no	no	yes	no	yes	yes	no	no	no	no	no	?	?
BBS75	right foot	no	yes	no	no	no	polycystic kidney dysplasia without functional	no	no	no	no	no	no	no	no	no	no	no	no	no	no	yes	no
BBS15	2 hands and 2 feet	yes	yes	unknown	no	no	kidney transplant at age 12	no	no	no		no	no	no	no	no		yes	no	no	no	no	no
BBS76	2 feet	yes	yes	no	no	no	no	no	no	no	no	no	no	no	no	no	no	no	no	no	no	yes	no
BBS82	2 hands and 2 feet	yes	yes	unknown	no	no	yes	no	yes	no		no	yes, until age 4	no	no	no		no	no	yes	yes	no	no
BBS72	2 hands and 2 feet	yes	yes	no	no	no	no	no	no	no	no	no	no	no	yes	yes	no	no	no	no	no	no	no
SRP95	one hand and 2 feet	no	yes	unknown	no	no	yes (stones)	no	no	no	no	no	no	no	no	no	no	no	no	no	no	no	no
BBS50	2 hands and 2 feet	yes	no	unknown	no	no	yes, stadium III	no	yes	no	no	no	no	no	no	no	no	no	no	no	no	no	no
BBS71	right foot	no	yes	no	no	no	no	no	no	no	no	no	yes, until age 4	no	yes	yes	no	no	no	yes	no	no	hypofunction
BBS86	no	no	yes	no	yes	no	no	no	no	no	no	no	no	no	yes	yes	no	no	no	no	no	no	no
BBS47	2 feet	no	no	unknown	no	no	no	no	yes	no	no	no	no	no	no	no	no	no	no	no	no	no	no
BBS57	2 hands and 2 feet	yes	yes since age 1	unknown	no	no	no	no	no	no	no	no	yes, until age 6	no	no	no	no	no	no	no	no	no	no
RCD768	left hand and left foot	no	no	no	no	no	no	no	no	no	no	no	no	no	no	no	no	no	no	no	no	no	no
**BBS12: 3 patients**																							
BBS59	right hand	no	no	no	no	no	no	no	no	no	no	no	no	no	no	no	no	no	no	no	no	yes	no
BBS58	2 hands and 2 feet	yes	yes, since age 7	unknown	no	no	no	no	no	no	no	no	no	no	no	no	no	no	no	no	no	no	no
BBS41	no	no	no	yes	yes	no	no	no	no	no	no	no	no	no	no	no	no	obstrictive bronchitis	no	no	no	no	no
**BBS16: 1 patient**																							
SRP890	no	no	no	no	no	liver hemangioma	yes since 2001	no	no	no	no	no	yes, until age 5	no	no	no	no	no	no	no	no	not known	no
