# Supplementary material for: Ophthalmic and Genetic Features of Bardet Biedl Syndrome in a German Cohort
Source: Genes (Basel). 2022 Jul 8;13(7):1218. doi: 10.3390/genes13071218 (PMC9322102; doi:10.3390/genes13071218)
Supplement: Supplementary file 1 [file genes-13-01218-s001.zip › Supplementary Tables S1-S3.pdf]

Supplementary Table S1: Genotypes of BBS patients in this study

Patient ID	Sex	Variant 1	Variant 2	Additional heterozygous variants	Segregation
BBS1: 15 patients					
BBS79	m	*BBS1* c.1169T>G;p.(M390R)	*BBS1* c.1169T>G;p.(M390R)	*MKKS* (BBS6) c.463C>T;p.(R155C)	-
BBS49	f	*BBS1* c.1169T>G;p.(M390R)	*BBS1* c.1169T>G;p.(M390R)	*ABCA4* c.5338>G;p.(P1780A)	-
BBS30	f	*BBS1* c.1169T>G;p.(M390R)	*BBS1* c.1169T>G;p.(M390R)		yes
SRP17	m	*BBS1* c.1169T>G;p.(M390R)	*BBS1* c.1169T>G;p.(M390R)	*PDE6B* c.1375G>A;p.(D459N)	-
BBS10-I	f	*BBS1* c.1169T>G;p.(M390R)	*BBS1* c.1169T>G;p.(M390R)		yes
BBS10-II	f	*BBS1* c.1169T>G;p.(M390R)	*BBS1* c.1169T>G;p.(M390R)		yes
BBS66	f	*BBS1* c.1169T>G;p.(M390R)	*BBS1* c.1169T>G;p.(M390R)	*ABCA4* c.2588G>C;p.(G863A)	-
BBS18	m	*BBS1* c.436C>T;p.R146*	*BBS1* c.1169T>G;p.(M390R)		-
BBS80	m	*BBS1* c.890G>A;p.R297Q	*BBS1* c.1169T>G;p.(M390R)		-
ARRP328	f	*BBS1* c.1169T>G;p.(M390R)	*BBS1* c.1232_1235delGAGG;<br>p.(G411Efs*12)		-
BBS87	f	*BBS1* c.1169T>G;p.(M390R)	*BBS1* c.1232_1235delGAGG;<br>p.(G411Efs*12)		-
BBS64	m	*BBS1* c.479G>A;p.(R160Q)	*BBS1* c.479G>A;p.(R160Q)	*RIMS1* c.3208G>A;p.(A1070T)<br>*GNAT2* c.427G>A;p.(A143T)<br>*ABCA4* c.3608G>A;p.(G1203Q)	yes
BBS78	m	*BBS1* c.479+4A>G;p.(?)	*BBS1* Deletion Exons 14-17		yes
BBS54	m	*BBS1* c.1570_1572delAAC;<br>p.(N524del)	*BBS1* c.1570_1572delAAC;<br>p.(N524del)		-
BBS77	m	*BBS1* c.784_793dup;<br>p.(N269Gfs*95)	*BBS1* c.1431_1447del;<br>p.(L478Rfs*17)		yes
BBS2: 3 patients					
BBS53	f	*BBS2* c.943C>T;p.(R315W)	*BBS2* c.943C>T;p.(R315W)		-
BBS35	m	*BBS2* c.661del;p.(L221Ffs*25)	*BBS2* c.1895G>C;p.(R632P)		-
ARRP379	m	*BBS2* c.413T>G;p.(I138S)	*BBS2* c.413T>G;p.(I138S)		-
BBS3: 3 patients					
BBS44-I	f	*ARL6* (BBS3)<br>c.291T>A;p.(S97R)	*ARL6* (BBS3) c.528G>T;<br>p.(W176C)		yes
BBS44-II	f	*ARL6* (BBS3)<br>c.291T>A;p.(S97R)	*ARL6* (BBS3) c.528G>T;<br>p.(W176C)		yes
BBS61	m	*ARL6* (BBS3)<br>Deletion Exons 4-9	*ARL6* (BBS3)<br>Deletion Exons 4-9		-
BBS4: 3 patients					
BBS37	m	*BBS4* c.322G>A;p.(A108T)	*BBS4* c.514dupA;<br>p.(I172Nfs*18)		-
BBS45	m	*BBS4* c.1103A>G;p.(D368G)	*BBS4* c.1103A>G;p.(D368G)		-
BBS55	m	*BBS4* c.157-3c>G;p.(?)	*BBS4* c.157-3c>G;p.(?)		-
BBS5: 3 patients					
BBS56-I	m	*BBS5* c.143-4_143-2ins400-<br>500;p.(?)<br>g.(170343574_170343578)<br>ins(400_500)	*BBS5* c.143-4_143-2ins400-<br>500;p.(?)<br>g.(170343574_170343578)<br>ins(400_500)	*BBS12* c.1139C>T;p.(T380I) hom	yes
BBS56-II	m	*BBS5* c.143-4_143-2ins400-<br>500;p.(?)<br>g.(170343574_170343578)<br>ins(400_500)	*BBS5* c.143-4_143-2ins400-<br>500;p.(?)<br>g.(170343574_170343578)<br>ins(400_500)	*BBS12* c.1139C>T;p.(T380I) hom	yes
BBS43	f	*BBS5* c.143-4_143-2ins400-<br>500;p.(?)<br>g.(170343574_170343578)<br>ins(400_500)	*BBS5* c.143-4_143-2ins400-<br>500;p.(?)<br>g.(170343574_170343578)<br>ins(400_500)		-
BBS6: 1 patient					
BBS33	m	*MKKS* (BBS6) c.110A>G;<br>p.(Y37C)	*MKKS* (BBS6) c.110A>G;<br>p.(Y37)		-
BBS7: 3 patients					
BBS70	f	*BBS7* c.712_715delAGAG;<br>p.(R238Efs*59)	*BBS7* c.968A>G;p.(H323R)		-
BBS62	f	*BBS7* c.968A>G;p.(H323R)	*BBS7* c.968A>G;p.(H323R)		-
BBS60	m	*BBS7* c.712_715delAGAG;<br>p.(R238Efs*59)	*BBS7* c.1037+29T>A;p.(?)		yes
BBS8: 1 patient					
BBS67	m	*TTC8* (BBS8) c.694G>A;<br>p.(G232R)	*TTC8* (BBS8) c.694G>A;<br>p.(G232R)		-
BBS9: 5 patients					
BBS40	m	*BBS9* c.263+1G>T;p.(?)	*BBS9* c.263+1G>T;p.(?)		-
BBS42-I	m	*BBS9* Deletion Exon 16	*BBS9* Deletion Exon 16	*SDCCAG8* (BBS16)<br>c.237T>A;p.(D79E)	yes
BBS42-II	m	*BBS9* Deletion Exon 16	*BBS9* Deletion Exon 16		yes
LCA70	f	*BBS9* c.1693+1G>A;p.(?)	*BBS9* c.1693+1G>A;p.(?)		yes
BBS74	f	*BBS9* Deletion Exons 7-8	*BBS9* Deletion Exons 7-8		yes
BBS10: 20 patients					
BBS81	m	*BBS10* c.271dup;p.(C91Lfs*5)	*BBS10* c.271dup;p.(C91Lfs*5)		-
BBS48	m	*BBS10* c.271dup;p.(C91Lfs*5)	*BBS10* c.271dup;p.(C91Lfs*5)	*MKKS* (BBS6) c.1363G>A;p.(E455K)	-
BBS46	f	*BBS10* c.271dup;p.(C91Lfs*5)	*BBS10* c.271dup;p.(C91Lfs*5)	*SDCCAG8* (BBS16)<br>c.1337G>A;p.(R446Q)	-

BBS16	m	*BBS10* c.271dup;p.(C91Lfs*5)	*BBS10* c.271dup;p.(C91Lfs*5)		yes
BBS83	f	*BBS10* c.271dup;p.(C91Lfs*5)	*BBS10* c.273C>G;p.(C91W)		yes
BBS84	f	*BBS10* c.271dup;p.(C91Lfs*5)	*BBS10* c.271dup;p.(C91Lfs*5)		-
BBS13	m	*BBS10* c.271dup;p.(C91Lfs*5)	*BBS10* c.271dup;p.(C91Lfs*5)		-
BBS85	f	*BBS10* c.271dup;p.(C91Lfs*5)	*BBS10* c.271dup;p.(C91Lfs*5)		-
BBS75	f	*BBS10* c.271dup;p.(C91Lfs*5)	*BBS10* c.530A>G;p.(Y177C)	*CEP164* c.4174C>T;p.(R1392W) *IFT122* c.668C>T;p.(P223L)	-
BBS15	m	*BBS10* c.271dup;p.(C91Lfs*5)	*BBS10* c.271dup;p.(C91Lfs*5)		-
BBS76	m	*BBS10* c.271dup;p.(C91Lfs*5)	*BBS10* c.235dupA; p.(T79Nfs*17)		-
BBS82	m	*BBS10* c.145C>T;p.(R49W)	*BBS10* c.271dup;p.(C91Lfs*5)		-
BBS72	f	*BBS10* c.145C>T;p.(R49W)	*BBS10* c.145C>T;p.(R49W)		-
SRP95	m	*BBS10* c.145C>T;p.(R49W)	*BBS10* c.145C>T;p.(R49W)		yes
BBS50	m	*BBS10* c.578>C;p.(L193S)	*BBS10* c.578>C;p.(L193S)		yes
BBS71	f	*BBS10* c.686C>T;p.(P229L)	*BBS10* c.271dup;p.(C91Lfs*5)		yes
BBS86	f	*BBS10* c.858_859dup;p.(Q287Lfs*1)2	*BBS10* c.901C>T;p.(L301V)		-
BBS47	m	*BBS10* c.931T>G;p.(S311A)	*BBS10* c.931T>G;p.(S311A)		yes
BBS57	f	*BBS10* c.1603_1606delGATT; p.(D535llefs*20)	*BBS10* c.1250C>T;p.(A417V)		-
RCD768	m	*BBS10* c.1802C>T;p.(P601L)	*BBS10* c.1802C>T;p.(P601L)		-
BBS12: 3 patients					
BBS59	f	*BBS12* c.1115_1116del;p.(F372*)	*BBS12* c.1237C>G;p.(L413V)		-
BBS58	m	*BBS12* c.2023C>T;p.(R675*)	*BBS12* c.1504>T;p.(A502S)		-
BBS41	f	*BBS12* c.898C>T;p.(Q300*)	*BBS12* c.1063C>T;p.(R355*)	*BBS4* c.65G>A;p.(R22Q)	-
BBS16: 1 patient					
SRP890	m	*SDCCAG8* (BBS16) c.740+356C>T; p.(?)	*SDCCAG8* (BBS16) c.740+356C>T; p.(?)		-

Footnote: [1]Genbank reference sequences: *ABCA4* NM_000350.3, *BBS1* NM_024649.5, *BBS2* NM_031885.5, *ARL6* (BBS3) NM_177976.3, *BBS4* NM_033028.5, *BBS5* NM_152384.3, *MKKS* (BBS6) NM_018848.3, *BBS7* NM_176824.3, *TTC8* (BBS8) NM_198309.3, *BBS9* NM_198428.3, *BBS10* NM_024685.4, *BBS12* NM_152618.3., *SDCCAG8* (BBS16) NM_006642.5, *CEP164* NM_014956.5, *GNAT2* NM_005272.5, *IFT122* NM_052985.4, *PDE6B* NM_000283.4, *RIMS1* NM_014989.5. Genomic position refers to GRCh37,hg19.

Supplementary Table S2: Mutation spectrum in BBS-related genes in this study cohort

Gene & Variant [1]	Reference (PMID)	Evidence[2]	Classification[3]	Splice prediction[4]	Allele count
BBS1<br>c.436C>T;p.(R146*)	PMID:32531858 [78]<br>same patient	PVS1, PM2, PP5 (ClinVar)	pathogenic		1
BBS1<br>c.479G>A;p.(R160Q)	PMID:26261414 [79]	PM2, PP3, PP5 (ClinVar)	likely pathogenic		2
BBS1<br>c.479+4A>G;p.(?)	this study	PM2, PP3	VUS	Splice AI: benign (low;0.85)<br>dbscSNV Ada: deleterious (1)<br>RF: deleterious (0.89)	1
BBS1<br>c.784_793dup;p.(N269Gfs*95)	this study	PVS1, PM2	likely pathogenic		1
BBS1<br>c.890G>A;p.(R297Q)	PMID:25170860 [37]	PM2, BP4	VUS		1
BBS1<br>c.1169T>G;p.(M390R	PMID:12118255 [80]	PS4, PM2, PP5 (ClinVar)	pathogenic		18
BBS1<br>c.1232_1235delGAGG;<br>p.(G411Efs*12)	PMID:32531858 [78]<br>same patient	PM2, PP5 (ClinVar)	pathogenic		2
BBS1<br>c.1431_1447del;p.(L478Rfs*17)	this study	PVS1, PM2	likely pathogenic		1
BBS1<br>c.1570_1572delAAC;<br>p.(N524del)	PMID:21344540 [81]	PM2, PM4	VUS		2
BBS1<br>Deletion Exons 14-17	this study	PVS1, PM2	pathogenic		1
BBS2<br>c.413T>G;p.(I138S)	PMID:32531858 [78]<br>same patient	PM2, PP5 (ClinVar)	likely pathogenic		2
BBS2<br>c.661del;p.(L221Ffs*25)	PMID:20120035 [82]	PVS1, PM2, PP5 (ClinVar)	pathogenic		1
BBS2<br>c.943C>T;p.(R315W)	PMID:11567139 [34]	PM2, PM1, PM5, PP3, PP5 (ClinVar)	pathogenic		2
BBS2<br>c.1895G>C;p.(R632P)	PMID:11567139 [34]	PM2, PP3, PP5 (ClinVar)	likely pathogenic		1
ARL6 (BBS3)<br>c.291T>A;p.(S97R)	PMID:32531858 [78]<br>same patient	PM2, PP2, PP3, PP5 (ClinVar)	VUS		2
ARL6 (BBS3)<br>c.528G>T;p.(W176C)	PMID:32531858 [78]<br>same patient	PM2, PP2, PP3, PP5 (ClinVar)	VUS		2
ARL6 (BBS3)<br>Deletion Exons 4-9	PMID:32531858 [78]<br>same patient	PVS1, PM2	pathogenic		2
BBS4<br>c.157-3C>G;p.(?)	PMID:32531858 [78]<br>same patient	PM2, PP3, PP5 (ClinVar)	likely pathogenic	SpliceAI: benign (low;0.78)<br>dbscSNV Ada: deleterious (0.99)<br>RF: deleterious (0.89)	2
BBS4<br>c.322G>A;p.(A108T)	PMID:23591405 [83]<br>same patient	PM2, PP5 (ClinVar)	likely pathogenic		1
BBS4<br>c.514dupA;p.(I172Nfs*18)	PMID:23591405 [83]<br>same patient	PVS1, PM2	likely pathogenic		1
BBS4<br>c.1103A>G;p.(D368G)	PMID:15666242 [84]	PM2, PP3, PP5 (ClinVar)	VUS		2
BBS5<br>c.143-4_143-2ins440;p.(?)<br>Intronic insertion of 400-500 bp<br>GRCh37 (hg19) NC_000002:<br>g.(170343574_170343578)<br>ins(400_500)	PMID:32531858 [78]<br>same patient,<br>additional family	PVS1, PM2	likely pathogenic	cannot be analyzed due to missing sequence information of the insertion	4
MKKS (BBS6)<br>c.110A>G;p.(Y37C)	PMID:10802661 [85]	PM2, PP3, PP5 (ClinVar)	pathogenic		2
BBS7<br>c.712_715delAGAG;<br>p.(R238Efs*59)	PMID:19402160 [86]	PVS1, PM2, PP5 (ClinVar)	pathogenic		2

BBS7<br>c.968A>G;p.(H323R)	PMID:12567324 [87]	PM2, PP5<br>(ClinVar)	likely<br>pathogenic		3
BBS7<br>c.1037+29T>A;p.(?)	this study	BS1, BP7	likely benign	Splice AI: no effect on splicing predicted (0.0/0.04)	1
TTC8 (BBS8)<br>c.694G>A;p.(G232R)	this study	PM2, PP3	VUS		2
BBS9<br>c.263+1G>T;p.(?)	PMID:32531858<br>same patient	PVS1, PM2	likely<br>pathogenic	SpliceAI: splice altering (1)<br>dbscSNV Ada: deleterious (1)<br>RF: deleterious (0.93)	2
BBS9<br>c.1693+1G>A;p.(?)	PMID:26766544 [88]<br>same patient	PVS1, PM2,<br>PP5<br>(ClinVar)	pathogenic	SpliceAI: splice altering (0.97)<br>dbscSNV Ada: deleterious (1)<br>RF: deleterious (0.94)	2
BBS9<br>Deletion Exons 7-8	this study	PVS1, PM2	pathogenic		2
BBS9<br>Deletion Exon 16	PMID:32531858 [78]<br>same patient	PVS1, PM2	pathogenic		4
BBS10<br>c.145C>T;p.(R49W)	PMID:16582908 [89]	PM2, PP5<br>(ClinVar)	likely<br>pathogenic		5
BBS10<br>c.235dup;p.(T79Nfs*17)	PMID:28143435 [90]	PVS1, PM2,<br>PP5<br>(ClinVar)	pathogenic		1
BBS10<br>c.271dup;p.(C91Lfs*5)	PMID:16582908<br>[89]v	PS4, PVS1,<br>PM2, PP5<br>(ClinVar)	pathogenic		27
BBS10<br>c.273C>G;p.(C91W)	PMID:16582908 [89]	PM2, PP5<br>(ClinVar)	likely<br>pathogenic		1
BBS10<br>c.530A>G;p.(Y177C)	PMID:19797195 [91]	PM2, PM1,<br>PP5<br>(ClinVar)	likely<br>pathogenic		1
BBS10<br>c.578T>C;p.(L193S)	PMID:32531858 [78]<br>same patient	PM2, PP3,<br>PM1, PP5<br>(ClinVar)	VUS		2
BBS10<br>c.686C>T;p.(P229L)	this study	PM2, PP5<br>(ClinVar)	likely<br>pathogenic		1
BBS10<br>c.858_859dup;p.(Q287Lfs*12)	this study	PVS1, PM2,<br>PP5<br>(ClinVar)	pathogenic		1
BBS10<br>c.901C>T;p.(L301V)	this study	PM2, BP7	VUS		1
BBS10<br>c.931T>G;p.(S311A)	PMID:16582908 [89]	PM2, PM1,<br>PP5<br>(ClinVar)	likely<br>pathogenic		2
BBS10<br>c.1250C>T;p.(A417V)	PMID:21052717 [92]	PM2, PP3,<br>PM1, PP5<br>(ClinVar)	likely<br>pathogenic		1
BBS10<br>c.1603_1606del; p.(D535llefs*20)	PMID:32531858 [78]<br>same patient	PVS1, PM2,<br>PP5<br>(ClinVar)	pathogenic		1
BBS10<br>c.1802C>T;p.(P601L)	this study	PM2, PP3,<br>PM1	VUS		2
BBS12<br>c.898C>T;p.(Q300*)	PMID:23591405 [83]<br>same patient	PVS1, PM2,<br>PP5<br>(ClinVar)	pathogenic		1
BBS12<br>c.1063C>T;p.(R355*)	PMID:17160889 [93]	PVS1, PM2,<br>PP5<br>(ClinVar)	pathogenic		1
BBS12<br>c.1115_1116del;p.(F372*)	PMID:17160889 [93]	PVS1, PM2,<br>PP5<br>(ClinVar)	pathogenic		1
BBS12<br>c.1237C>G;p.(L413V)	PMID:32531858 [78]<br>same patient	PM2, PP5<br>(ClinVar)	likely<br>pathogenic		1
BBS12<br>c.1504>T;p.(A502S)	PMID:32531858 [78]<br>same patient	PM2, PM1,<br>PP5<br>(ClinVar)	likely<br>pathogenic		1
BBS12<br>c.2023C>T;p.(R675*)	PMID:20827784 [94]	PVS1, PM2,<br>PP5<br>(ClinVar)	pathogenic		1
SDCCAG8 (BBS16)<br>c.740+356C>T;p.(?)<br>GRCh37 (hg19)<br>NC_000001.10:g.243468435C>T	PMID:20835237 [95]	PM2, PP5<br>(ClinVar)	likely<br>pathogenic	Splice AI: no effect on splicing predicted (0.0/0.04), but missplicing confirmed by RT-PCR (PMID: **20835237)**	2

Footnote: [1]Genbank reference sequences: *BBS1* NM_024649.5, *BBS2* NM_031885.5, *ARL6* (BBS3) NM_177976.3, *BBS4* NM_033028.5, *BBS5* NM_152384.3, *MKKS* (BBS6) NM_018848.3, *BBS7* NM_176824.3, *TTC8* (BBS8) NM_198309.3, *BBS9* NM_198428.3, *BBS10* NM_024685.4, *BBS12* NM_152618.3., *SDCCAG8* (BBS16) NM_006642.5. Genomic position refers to GRCh37,hg19.

[2,3] Evidence and classification refers to the standards and guidelines provided by the American College of Medical Genetics and Genomics (ACMG) and the Association for Molecular Pathology to classify the identified variants [96] [1]. VUS - variant of uncertain significance; PVS1 - pathogenic very strong (Null variant in a gene where loss of function is a known mechanism of disease); PM1 - pathogenic moderate (Non-truncating non-synonymous variant is located in a mutational hot spot and/or critical and well-established functional domain); PM2 - pathogenic moderate (Extremely low frequency in gnomAD population databases); PM5 - pathogenic supporting (Different amino acid change as a known pathogenic variant); PP2 - pathogenic supporting (Missense variant in a gene with low rate of benign missense mutations and for which missense mutation is a common mechanism of a disease); PP3 - pathogenic supporting (For a missense or a splicing region variant, computational prediction tools unanimously support a deleterious effect on the gene); PP5 - pathogenic supporting (Reputable source recently reports variant as pathogenic, but the evidence is not available to the laboratory to perform an independent evaluation, e.g. ClinVar); PS4 - pathogenic strong (Typical case control studies with an OR; or, multiple unrelated probands with consistent phenotypes).
[4]SpliceAI uses deep neural networks to predict whether splicing events occur. The score can range from 0 to 1, when scores can be interpreted as the probability of the variant being splice-altering. dbscSNV Ada predicts for SNVs within splicing consensus regions (−3 to +8 at the 5' splice site and −12 to +2 at the 3' splice site), their potential of altering splicing by using ensemble score computed using AdaBoost algorithm on the outputs of several other prediction tools. The score can range from 0 to 1, when higher values are more likely of being deleterious. RF predicts for SNVs within splicing consensus regions (−3 to +8 at the 5' splice site and −12 to +2 at the 3' splice site), their potential of altering splicing by using ensemble score computed using Random Forest algorithm on the outputs of several other prediction tools. The score can range from 0 to 1, when higher values are more likely of being deleterious.

Supplementary Table S3: Classification of additional heterozygous variants in BBS- or IRD-related genes.

Gene & Variant [1]	Reference (PMID)	Evidence[2]	Classification[3]	Splice prediction[4]
ABCA4 c.2588G>C;p.(G863A)	PMID: 9054934 [97]	PS4, PM2, PP3, PP2, PP5	pathogenic	Revel Deleterious<br>dbscSNV Deleterious<br>Splice AI Splice-altering
ABCA4 c.3608G>A;p.(G1203Q)	PMID: 15192030 [98]	PM2, PP2, PP5	VUS	Revel Deleterious (Low)<br>dbscSNV Deleterious<br>Splice AI Splice-altering (Low)
ABCA4 c.5338>G;p.(P1780A)	PMID: 10746567 [99]	PP5, PM2, PM1, PP3, PP2	pathogenic	Revel Deleterious<br>MetaLR Deleterious<br>Splice AI Benign
BBS4 c.65G>A;p.(R22Q)	this study	PM2	VUS	no missplicing predicted
BBS12 c.1139C>T;p.(T380I)	this study	Criteria unmet	VUS	no missplicing predicted
CEP164 c.4174C>T;p.(R1392W)	this study	PM2	VUS	no missplicing predicted
GNAT2 c.427G>A;p.(A143T)	this study	PM2	VUS	Revel Deleterious (Low)<br>MetaLR Deleterious<br>Splice AI Benign
IFT122 c.668C>T;p.(P223L)	this study	PM2	VUS	Revel Benign (Low)<br>MetaLR Deleterious<br>Splice AI Benign
MKKS (BBS6) c.1363G>A;p.(E455K)	this study	PM2	VUS	Revel Benign (Low)<br>MetaLR Deleterious (Low)<br>Splice AI Benign
MKKS (BBS6) c.463C>T;p.(R155C)	this study	PM2	VUS	Revel Benign (Low)<br>MetaLR Deleterious (Low)<br>Splice AI Benign
PDE6B c.1375G>A;p.(D459N)	this study	PM2	VUS	no missplicing predicted
RIMS1 c.3208G>A;p.(A1070T)	this study	PM2	VUS	no missplicing predicted
SDCCAG8 (BBS16) c.1337G>A;p.(R446Q)	this study	PM2	VUS	no missplicing predicted
SDCCAG8 (BBS16) c.237T>A;p.(D79E)	this study	PM2	VUS	no missplicing predicted

Footnote: [1]Genbank reference sequences: *ABCA4* NM_000350.3, *CEP164* NM_014956.5, *GNAT2* NM_005272.5, *IFT122* NM_052985.4, *PDE6B* NM_000283.4, *RIMS1* NM_014989.5, *BBS4* NM_033028.5, *MKKS* (BBS6) NM_018848.3, *BBS12* NM_152618.3., *SDCCAG8* (BBS16) NM_006642.5.

[2,3] Evidence and classification refers to the standards and guidelines provided by the American College of Medical Genetics and Genomics (ACMG) and the Association for Molecular Pathology to classify the identified variants [96]. VUS - variant of uncertain significance; PM1 - pathogenic moderate (Non-truncating non-synonymous variant is located in a mutational hot spot and/or critical and well-established functional domain); PM2 - pathogenic moderate (Extremely low frequency in gnomAD population databases); PP2 - pathogenic supporting (Missense variant in a gene with low rate of benign missense mutations and for which missense mutation is a common mechanism of a disease); PP3 - pathogenic supporting (For a missense or a splicing region variant, computational prediction tools unanimously support a deleterious effect on the gene); PP5 - pathogenic supporting (Reputable source recently reports variant as pathogenic, but the evidence is not available to the laboratory to perform an independent evaluation, e.g. ClinVar); PS4 - pathogenic strong (Typical case control studies with an OR; or, multiple unrelated probands with consistent phenotypes).

[4]SpliceAI uses deep neural networks to predict whether splicing events occur. The score can range from 0 to 1, when scores can be interpreted as the probability of the variant being splice-altering. dbscSNV Ada predicts for SNVs within splicing consensus regions (−3 to +8 at the 5’ splice site and −12 to +2 at the 3’ splice site), their potential of altering splicing by using ensemble score computed using AdaBoost algorithm on the outputs of several other prediction tools. The score can range from 0 to 1, when higher values are more likely of being deleterious. RF predicts for SNVs within splicing consensus regions (−3 to +8 at the 5’ splice site and −12 to +2 at the 3’ splice site), their potential of altering splicing by using ensemble score computed using Random Forest algorithm on the outputs of several other prediction tools. The score can range from 0 to 1, when higher values are more likely of being deleterious.
